# Supplementary material for: Are carbon emissions trading and green financial instruments synergistic? -Comprehensive quantitative research based on content analysis
Source: PLoS One. 2024 Mar 7;19(3):e0298601. doi: 10.1371/journal.pone.0298601 (PMC10919601; doi:10.1371/journal.pone.0298601)
Supplement: S1 Data — (ZIP) [file pone.0298601.s001.zip › Data/地级市Z_F工作报告.zip!/╣π╢1⁄2/╓╨╔╜╩╨/└┤╫╘╣1⁄2╓┌║┼í╛┬φ┐╦╩2╛▌═°í┐_2014.docx]

2013年工作回顾

2013年，我市全面贯彻落实党的十八大和习近平总书记视察广东重要讲话精神，沉着应对复杂形势，顽强拼搏、奋发有为，经济社会发展取得了重要成绩。在省委、省政府和市委的正确领导下，在市人大和市政协的监督支持下，市政府团结全市人民和海内外乡亲，大兴实干之风，以创新发展为动力，以“三重两大一保障”为重点带动全面工作，以转型升级为路径优化经济发展，以改善民生为目的提高市民福祉，着力提高工作的前瞻性、针对性和有效性，实现了有质量的稳定增长、可持续的全面发展，较好地完成了市十四届人大三次会议确定的目标任务，统筹推进“五位一体”建设取得新成效。

经济发展在应对挑战中稳中向好。全市实现生产总值2638.9亿元，比上年增长10%。人均生产总值8.34万元，增长9.4%。工业增加值1404.2亿元，增长11.6%。服务业增加值1108.4亿元，增长9%。公共财政预算收入225.3亿元，增长11.6%。固定资产投资962.9亿元，增长15.2%。社会消费品零售总额890.6亿元，增长10.4%。出口总值264.8亿美元，增长7.5%。实际利用外资6.46亿美元。开拓新兴市场取得突破。实施《珠三角规划纲要》成效明显，全部考评指标完成省的任务，其中5项指标排全省前列。成功举办海峡两岸中山论坛、粤澳合作联席会议，粤港澳合作考核排全省第3。

质量效益在转型升级中不断提高。三次产业结构调整为2.5：55.5：42，轻重工业比为54.7：45.3。规模以上工业企业利润增长15.8%。“新三百”培育企业产值增速高出全市平均水平5.7个百分点，百亿级企业增至8家，十亿级企业增至98家，百亿级产业集群达9个。1.9万户个体户转型升级为企业。新增中环广场、小榄金融商务区两个省级现代服务业集聚区。建成国家标准化生产示范园3个、省级现代农业园区6个。单位建设用地二三产业增加值、平均立项投资额分别上升6.35%、29.7%。先进制造业、高技术制造业增加值分别占规模以上工业增加值的35.4%和15.7%。加工贸易“委托设计+自主品牌”生产方式比重提高10个百分点，一般贸易、机电产品出口比重分别提高1.9和1.8个百分点，服务贸易增长225%。金融业增加值增长13%，境内外上市公司达23家。全年培养和引进各类人才5万多人，增长18.6%。全市研发经费占生产总值比重2.28%，省科技奖特等奖、省发明专利金奖实现“零”的突破，城市综合创新能力跻身全国地级市第5。

城乡建设在统筹推进中更加协调。完成“十二五”规划中期评估调整，编制了主体功能区规划。翠亨新区进入实质开发建设阶段，起步区基础设施建设加快推进。制定“十二五”后三年重要基础设施建设方案。深中通道上报国家立项，广中江高速中山段动工，东部外环高速、中开高速中山段及小榄支线纳入省高速公路建设计划和省重点项目，中环路建成通车，古神公路二期、十水线、翠亨快线、105国道改造等工程进展顺利，黄圃港区多用途码头建成运营。全市19条断头路已接通14条，完成70座危桥改造。公路密度每百平方公里达128公里。实现公交“一盘棋”。有序开展闲置用地处置，成为省统筹城乡土地综合整治试点，获得省节约集约用地考核二等奖、“三旧”改造考核二等奖。

环境保护在生态建设中不断加强。加强水体治理，雨污分流城区工程提前完成年度任务，镇区工程加紧推进。启动实施“南粤水更清”行动计划。建立重金属污染物排放总量前置审核制度，开展二氧化硫、碳排放等主要污染物综合防治。实现镇区垃圾转运站全覆盖。秀美村庄建设有序推进，投入13.5亿元建设783个农村项目。三年累计投入37亿元，“绿化大提升”行动计划圆满完成。万元生产总值能耗下降3.9%，主要污染物总量减排、环境保护责任考核分获全省第1和第2。

民生福祉在群众生活中稳步提高。财政民生支出163.3亿元，占财政支出68.8%。城镇居民人均可支配收入34274元，农村居民人均纯收入21727元，分别增长10.1%和12.3%。向社会承诺的十件民生实事圆满完成。城镇登记失业率控制在2.3%以下，新增就业5万人，农村劳动力技能培训转移就业考核全省第1。建成社区就业服务基地186个，在全省率先建立高校毕业生创业农业孵化基地。社会福利普惠水平提升，最低生活保障标准提高11.6%，改造双低家庭和优抚对象危房500户。制定困难居民重特大疾病医疗救助办法。完成新社会福利院首期工程，新建5个居家养老服务中心，为近800名孤寡老人提供免费居家养老上门服务。基本建成各类保障性住房3705套，新开工建设2968套。建成平价商店203家。实现学前三年行动计划目标，完成中小学校长职级制改革试点。高考录取率连续八年全省第1，职业教育整体水平处于全省前列。出台“1+N”系列医改政策，公共卫生服务、区域卫生信息化等工作稳步推进，新增4个国家卫生镇。公共文化服务体系逐步健全，文化场馆免费服务群众373万人次。中山籍运动员参加第12届全运会获3金2银1铜。扶贫工作巩固提升，对口帮扶潮州工作开局良好。

社会环境在综合治理中和谐稳定。出台社会建设中长期规划。扎实推进全民修身行动“巩固年”工作，中山在全国文明城市测评中位居地级市前列。举办首届“博爱100”公益创投活动，设立5000万元社会管理创新专项资金。积分制管理获中国地方政府创新奖，超万名异地务工人员及其子女通过积分入户入学。率先推行村（居）委会特别委员制度。开展律师法律服务进村（居）活动，律师与村（居）签约服务覆盖率达100%。人口计生服务管理不断优化。出台政府向社会组织购买服务目录。新增社会组织163家，持证社工人数超过1800人。实施“全民治安”工程，全市110有效警情下降26.4%。成功创建“无医闹城市”，“全民禁毒”经验向全国推广，连续5次荣获“全国社会管理综合治理优秀地市”称号，成为全省唯一连续3次获得“长安杯”的城市。化解一批信访历史积案，100%完成省交办的任务。深化数字城管系统应用，市容市貌管理不断加强。食品药品安全进社区工作加快推进。全面排查整治安全生产隐患。应急管理水平不断提高。台务、双拥、统计、审计、民族宗教、国防建设、慈善事业、妇女儿童、老龄、残疾人、气象、人防、档案、地方志等各项工作取得新成绩。

过去一年，我们始终注重加强政府自身建设。办理人大代表建议132件、政协提案190件，满意和基本满意率达100%。积极应对复杂形势，出台实施“新三百”计划、推进“三重”建设、促进企业兼并重组、优化营商环境等一揽子惠企政策，实施口岸延时通关，解决企业实际困难。加强网络问政，深化政务公开，网上办事大厅中山分厅建设走在全省前列。新建社区综合服务大厅200个。严格落实中央八项规定，深入整治“四风”和庸懒散奢等不良风气。严格政府绩效管理，实现公务经费零增长，预算追加经费联审核减60%。启动商事登记制度改革，完成第六轮行政审批制度改革，理顺食品药品监管机构、卫生和计划生育机构设置。建立市公共资源交易中心，建成农村集体资产监管和交易平台。行政复议工作有力地促进了政府依法行政。

各位代表！回顾过去一年，我们深感成绩来之不易。这是省委、省政府和市委正确领导的结果，是市人大及其常委会和市政协监督支持的结果，是全市干部群众团结奋斗的结果，是海内外各界关心帮助的结果。在此，我代表市政府，向全市广大工人、农民、知识分子、企业家、干部群众，向各民主党派、工商联、各人民团体、无党派人士和社会各界人士，向驻我市解放军和武警官兵、人民警察，向所有关心支持中山发展的老领导、老同志、港澳台同胞、海外侨胞和国内外友人表示衷心感谢！

我们也清醒地认识到，我市发展还存在一些困难和问题，主要是：制约发展的体制机制问题依旧存在，长期积累的矛盾相互交织，增加了发展难度；项目落地难、发展后劲不足等问题没有根本解决；内需乏力更加突出，经济增长的压力越来越大；面临城市升级和产业转型的双重压力，粗放型增长的路径依赖较为严重，以城带产、产城融合的步伐亟待加快；空气污染、交通拥堵等市民关切的问题比较突出，保障和改善民生的任务任重道远；社会治理水平有待提高，维稳压力加大；部分干部不敢担当，开创性不够，研究新情况劲头不足，解决新问题办法不多等。对此，我们将高度重视，采取有力措施，认真加以解决。

2014年工作安排

各位代表！2014年是深入贯彻党的十八届三中全会精神的关键一年，也是我市的“深化改革年、项目落实年、效能提升年”。综观当前宏观经济形势，虽然经济下行的压力较大，但仍有许多有利条件，我们仍然可以大有作为。十八届三中全会绘就了深化改革的路线图，胡春华书记寄语我市走创新驱动的工业化之路，为中山发展指明了方向。我们要在中央顶层设计基础上，把握改革的窗口期，深入贯彻市委《关于全面深化改革增创发展新优势的实施意见》，落实《2014年重点领域和关键环节改革要点》，从最紧迫的事项改起，从人民群众最期盼的领域改起，从制约经济社会发展最突出的问题改起，从社会各界能够达成共识的环节改起，着重处理好五方面关系：市场与政府的关系，破除体制障碍与健全制度建设的关系，整体推进与重点突破的关系，市域统筹与镇区活力的关系，顶层设计与基层首创的关系。让改革创新激发中山“二次创业”的蓬勃生机，开创我市改革开放新局面。

2014年政府工作的总体要求是：全面贯彻落实党的十八大、十八届三中全会、中央经济工作会议和省委十一届三次全会精神，按照市委十三届五次全会的部署，坚持稳中求进的总基调，把改革创新贯穿于经济社会发展各个领域各个环节，推进制度创新与发展模式创新，推进城市升级与产业升级，推进社会和谐与城乡和谐，强化生态基底与民生保障，努力保持经济社会持续健康发展。

2014年经济社会发展的主要预期目标是：生产总值增长10%，固定资产投资增长13%，社会消费品零售总额增长9%，出口总值增长6%，公共财政预算收入增长11%，城乡居民收入增长与经济增长基本同步，居民消费价格涨幅控制在3.5%左右，城镇登记失业率控制在3.5%以内，人口自然增长率控制在8‰以下，万元生产总值能耗下降3.8%，节能减排约束性指标完成省下达年度计划。

为实现上述目标，必须做好以下工作。

一、加快转型升级，实现经济持续健康发展

加快转型升级，既要把根牢牢地扎在实体经济的土壤上，优化存量、提升增量，不断创新发展模式，更要充分激发内外两个市场活力，打造中山经济升级版。

振兴实体经济。加大市级对土地、环境、水电等资源要素的统筹力度，优先向实体经济倾斜。做强先进制造业，重点发展游艇、风电、光电等装备制造业，推进神湾游艇产业服务集聚区、粤新海洋工程装备制造基地等项目建设。做大战略性新兴产业，重点发展高端新型电子信息和健康医药产业，加快北斗物联网产业基地、健康产业“一基地、三园区”等项目建设。做优传统产业，建设大涌镇产业转型升级试点。加快发展现代服务业，着力发展金融、物流、文化创意、工业设计等服务业，推进服务业集聚区建设，加快紫马奔腾、金鹰广场、海雅广场等项目进度。

优化存量。整合44个工业园资源，完善产业发展载体，促进产业园区就地升级。鼓励企业加强技改、更新设备、增资扩产。鼓励企业做大做强做优，年内新增规上限上企业360家。鼓励企业兼并重组，培养一批具有较强竞争能力的大型企业集团。落实“新三百”计划，发展“三自一资”企业。鼓励支持中小微企业繁荣发展，完善中小微企业综合服务体系，培育壮大科技型、创新型中小企业。实施品牌标准专利战略，提升企业产品质量。鼓励创新商业模式，发展移动电商、线上线下相结合的新型业态，促进实体经营与网上销售、快速物流、消费金融相结合；加快制造业服务环节分离发展服务业；通过提升传统产业文化创意水平，提高产品附加值。依法依规做好第三次全国经济普查工作。

提升增量。促进投资合理增长，优化投资结构。今年安排市重点项目94项，年度计划投资292.9亿元。新增用地指标向重点项目倾斜，提高单位土地产出率，提高经济密度和空间活力。着力破解征地拆迁、立项审批、资金紧缺等难题。落实好创新招商引资纲领，组织开展光电等产业招商行动，推进健康医药产业招商项目落地建设。健全项目流转超市管理。大力发展总部经济，利用域外资源发展壮大本土企业。

扩大内需。激发企业创业活力。推动中山产品全国行、网上行。大力发展电子商务，鼓励企业上网触电。加快推广“中山美居”，推动电子商务与优势产业融合发展。研究出台促进信息消费实施方案，加快发展医疗保健、养老服务、家政服务、教育培训等服务业态。办好中山休闲旅游文化节，启动中山美食“五个一”工程。开展灯饰游、游艇游、食品游、红木文化游，培育产业旅游市场。引导房地产健康发展，科学安排城市综合体的布局和开发节奏。

做强外贸。优化外贸环境，力促出口，加大进口。积极参与21世纪海上丝绸之路建设，开发东盟、南亚、非洲、南美、中东、俄罗斯等新兴市场，巩固欧美等传统市场。组织参加国际重点专业展会，引导企业“走出去”建立产销网络。加大出口信用保险支持力度，加强贸易摩擦应诉指导。打造乳制品、红酒、木材等进口交易平台，扩大资源和消费品进口。争取落实中山港向外轮开放。加快电子口岸建设，推行“一次申报、一次查验、一次放行”的快速通关模式，发展跨境电子商务。

二、释放要素活力，打造更加优良的营商环境

营商环境最能体现一个地区的综合竞争力。我们要发展，就必须在营商环境上下功夫，要比别的城市更加开放灵活，更能聚财留人，努力成为全国综合营商成本最低的城市之一。

深化行政管理体制改革。通过改革，还权于市场、放权于企业、分权于中介，让社会感觉到干部作风在一天天好转、办事效率在一天天提高、营商环境在一天天优化。精简行政审批，在完成专业技术评审、项目招投标及报批资料完备的前提下，社会投资项目审批时限缩短到55个工作日以内，政府投资项目缩短到75个工作日以内。加快建立行政服务中心，构建网上办事大厅与实体办事大厅相结合的行政服务体系。全面实施商事登记制度改革，建立“宽进严管”的准入体系。深化简政强镇事权改革，加快制定市镇权责清单，市场机制能有效调节的经济活动一律取消审批，由镇政府管理更为便利有效的审批事项一律下放。稳步推进行政执法体制改革，完善综合执法和联合执法机制。逐步建立事业单位法人治理结构，加大政府购买服务力度。

深化经济领域改革。全面清理与改革不相适应的各类文件，该废止的废止，该修改的修改。建立负面清单管理方式，消除民间投资、外商投资的隐性准入障碍。鼓励各类经济主体公平参与市场竞争，建设省企业投资备案制度改革试点。深化财税管理体制改革，探索建立事权与支出责任相适应的制度。深化产业扶持政策改革，安排产业扶持资金6亿元，比上年净增1亿元。深入推进市属国有企业改革，发展交叉持股的混合所有制经济，实行国有资本独立经营预算，探索组建市属国有资本运营公司，规范镇属国有资产管理。深化公共资源交易体制改革，完善公共资源交易平台。开展资源性产品、准公共产品等领域的价格改革。继续推进社会信用体系和市场监管体系建设。

优化金融服务。加快城乡金融服务一体化综合改革试点建设。培育发展地方金融，发展村镇银行、小额贷款公司、融资担保公司，探索组建民营银行，推进“五区一平台”建设。加强专业镇金融服务中心建设，搭建中小微企业与银行的对接平台。加快发展社区金融、物流金融和跨境金融服务。推进企业境内外上市和新三板挂牌。鼓励企业增发股权、发行债券，引导企业进入区域股权交易平台。加强金融生态建设，防范和化解金融风险。

加强科技创新。完善创新创业公共服务体系，推进创新链、产业链、资金链“三链融合”。鼓励协同创新，集聚重点高校、科研院所和龙头企业的创新资源，开展重点科技攻关。探索建立全市生产力促进中心联盟，完善市级生产力促进中心功能。深化科技管理改革，探索建立主要由市场决定技术创新项目、经费分配和评价成果的机制。深化科技金融创新，推广知识产权质押、专利保险，引导金融资本进入科技创新和高科技产业领域。加快建设国家知识产权示范城市，严厉打击侵犯知识产权的违法行为。

汇聚优秀人才。安排4200万元，用于人才建设。清除本科及以上人才落户障碍，简化人才落户手续。优化创新科研团队创业环境，加大领军人才、紧缺人才和高技能人才的引进力度，深化“名师名医名家培育工程”。充分利用省部共建国家级留学人员创业园的契机，引进一批高层次人才和高水平科研机构。办好高层次人才联谊会，将招才引智洽谈会打造成全国知名人才节。

深化区域合作。实施《珠三角规划纲要》“九年大跨越”工作方案。加强珠中江经济圈基础设施建设、生态环保、公共服务等领域的合作。深化与港澳台合作，推进港澳参与翠亨新区开发，加快实现中山澳门游艇自由行。推动与港澳地区服务贸易自由化，吸引港澳资本发展建筑设计、会计审计等现代服务业。

三、统筹资源管理，推动城乡建设升级

建设精品城市，以城市功能完善带动转型升级。注重规划引领，统筹城乡管理，高效用好土地资源，让生产空间更集约充分，让生活空间更宜居适度。

改革规划管理体制。通过改革，优化和理顺规划、国土管理政策，让城乡规划、建设和管理更能有效对接。树立规划先行和全域中山的理念，开展城市总体规划修编，加快推动“多规合一”。健全城市空间发展权转移机制，促进旧城改造与新区建设联动，探索容积率异地转移，进入公共资源交易平台。

统筹土地使用管理。实行严格的土地用途管制，发挥规划对土地的宏观调控作用。强化土地统一管理，加强全市土地规模、指标及供应计划的统筹，逐步建立城乡一体的建设用地市场。加大闲置土地处置力度，盘活存量建设用地。加快“三旧”改造，鼓励连片开发。强化用地转功能、容积率调整、公建配套规划管控政策，鼓励建设高层现代厂房，全面实施经营性用地按楼面地价竞价出让。落实城乡建设用地增减挂钩政策。

加快提质扩容步伐。构建全市“一主两副”和中心城区“一城双核”空间格局，适度拉大框架，提升城市承载力。全面推进主体功能区规划实施。开展东部地区发展规划，推动东部城镇融入宜居湾区建设。划定市域生态控制线，开展河海岸线规划。实施水系、绿道连通工程。完善历史文化名城保护规划体系，连片打造烟墩山、西山寺、中山纪念堂文化轴线，保护提升从善坊历史文化街区。加强城市设计、街区设计和重要建筑设计。推进市政管网、水利等基础设施建设，加快输变电工程、天然气应急门站等能源项目建设。

加快秀美村庄建设。安排1亿元，用于秀美村庄建设。加强农村环境综合整治，加大基础设施建设力度，推进生活设施配套、乡村景观改造、田园风光维护、绿化美化建设。推进公共服务进村入户，提升农村社会发展水平，打造“宜居、宜业、宜游”的秀美村庄。制定秀美村庄建设评价指标体系，指导镇村开展创建工作，年内验收一批秀美村庄创建单位。

加快建设发展平台。安排5.5亿元，推动翠亨新区起步区建设。紧扣基础设施建设、公共服务配套和标杆性项目落户，加快翠亨新区先进智造区、现代服务业集聚区、中瑞工业园与文化园区规划建设，推进中山港东移。用地指标优先向翠亨新区倾斜。加快广珠城轨站场综合开发，实现岐江新城和中山北站、中山站“一新城两枢纽”区域联动发展。

加快发展交通事业。实施综合交通规划，统筹安排35亿元用于交通建设。优化深茂铁路线路及站场方案，开展广中珠澳城轨选线工作。加快高速公路和干线路网建设，推动深中通道、中开高速及小榄支线前期工作，推进广中江高速建设。加快“改造一环、建设二环、规划三环”计划，完成105国道跨线桥改造工程，动工建设二环快速路东段，加快翠亨快线、广珠中线二期、中山港大桥扩建、横四线西段、纵四线黄圃至东升段、大南公路等工程进度。全面打通重点断头路。推动公交优先发展，建设省公共交通示范城市。实施道路交通精细化管理。

四、创新社会治理，营造和谐安定的社会环境

着眼于维护广大市民的根本利益，最大限度地增加和谐因素，激发社会活力，提高社会治理水平，努力营造包容和谐、公平正义、守望相助的社会氛围，让人民安居乐业，让社会安定有序。

改进社会治理方式。通过改革，弥补社会治理领域手段短缺、方式滞后的短板，让社会环境既充满活力又和谐有序。建设社区公共服务信息平台，实现社区综合服务大厅全覆盖。推动“2+8+N”模式向城市社区延伸。扩宽新中山人参与社会治理渠道。做好村级换届选举工作，确保实现“两个80%”的目标。完善社会动员机制，动员企业和社会力量，积极参与社会建设和管理。实施直接登记、分类登记和等级评估，培育壮大社会组织，推动去行政化、去垄断化。加快枢纽型社会组织体系建设，探索建立粤港澳社工义工合作试验区。

做好矛盾纠纷化解工作。构建公共法律服务体系，打造半小时法律服务圈。健全大调解工作格局，畅通和规范群众诉求表达、利益协调、权益保障渠道，使群众诉求能反映、问题能解决、情绪可排解、权益有保障。加强社情民意的收集和研判，推行重大决策社会稳定风险评估。健全矛盾化解、信访处置机制，促进劳动关系和谐。加强媒体和网络舆论引导，凝聚社会正能量。

深化“平安中山”建设。基本实现平安细胞创建目标，全面实现“村村通视频”。完善突发事件应急处置机制。推进气象现代化建设，加强防灾减灾和灾害治理。做好安全生产工作。更加注重保障“舌尖上的安全”，安排6500万元用于食品检验检测，实施食品生产源头治理和产销全程监管，让人民吃得好、吃得放心。

五、加强生态文明建设，打造宜居舒适的美丽中山

强化生态基底，把生态文明建设放在更加突出的位置，绝不以牺牲环境为代价换取经济增长，让城市融入大自然，让市民望得见蓝天白云、看得见青山绿水、呼吸到新鲜空气。

推进环境治理改革。综合运用市场手段和行政手段，修复和保护生态环境。加快生态文明城市建设，创建生态示范村（社区）。严格执行新建扩建项目污染物前置审核制度。推广合同能源管理模式。培育环保市场，探索开展节能量、碳排放权、水权交易试点，启动排污权有偿使用和交易试点工作，初步完成排污权初始分配。探索推进环境污染第三方治理。完善生态补偿机制。

发展现代生态农业。实施现代农业发展三年行动计划，打造优质高效、绿色安全的现代都市农业。加快高标准基本农田建设，安排3400万元，启动主干农路硬底化二期工程。建设现代农业园区，发展设施农业，完善现代农产品流通体系。推广新技术，引进新品种，发展“三品一标一名牌”产品，加快室内循环水养殖项目建设。探索进一步扩大农业保险的覆盖面。发展家庭农场和现代农场，鼓励适度规模经营。牢牢端稳自己的饭碗，提高粮食安全保障水平。加强农产品质量安全监管，落实重大动植物疫病防控措施。鼓励大学生开展现代农业创业。

实施绿化中山大行动。推进“135绿色战略”，创建国家森林城市。安排3.2亿元，用于绿化和公园建设。推进森林进城围城、村庄绿化美化、全民修身绿化“三大工程”，重点建设道路沿线、一河两岸、滨海廊道等绿色景观带，构建市镇村、山河海为一体的绿色景观网络。动工建设五马峰公园，改造紫马岭公园、中山公园，建成一批森林公园和湿地公园。

开展水环境综合治理。统筹安排11亿元，用于内河整治。落实“南粤水更清”行动计划。坚持质量第一，完善工程设计，加强施工管理，推进岐江河流域雨污分流工程，建设分散型污水处理设施及配套管网。落实河段长责任，实施最严格水资源管理制度，抓好用水“三条红线”管理。加快推进抗咸工程。

大力推进节能减排。用最严厉的措施、最严格的监管，推进节能减排。严格环境准入和污染物排放标准，实行项目环评区域限批、挂牌督办、限期整改。淘汰落后产能，推进工业、建筑、交通、公共机构等领域节能。加强工业企业生态化改造，创建一批资源节约、环境友好示范企业。实行阶梯电价、水价、气价，倒逼企业节能减排。倡导绿色生活，完善城乡慢行系统。

六、增进民生福祉，努力让市民过上更好生活

强化民生保障，既要注重民生政策的普惠性和公平性，更要突出重点、兜住底线，让民生工作每年都有看得见的进步、感受得到的实惠，让市民更幸福、城市更包容、社会更温暖。

改进民生服务供给方式。通过改革，释放民生建设领域活力，建立以政府投入为主导、社会力量多元参与的新格局。改变民生实事由政府包办的做法，实行“市民点菜、政府配餐”，让政府的供给与市民的期盼相统一，让民生工程真正成为民心实事。

完善社会保障体系。统筹安排8.6亿元，用于社会保障事业。健全就业创业服务体系，完善社会保险制度。创新社会救助方式方法，提升应对自然灾害及突发事件的能力。发挥社会救助网上协同平台作用，加大困难群体患重特大疾病的医疗救助力度。实施双低家庭和优抚对象危房改造工程，扩大住房保障范围。推进慈善、残疾人、红十字等事业改革发展。

增加城乡居民收入。健全工资正常增长机制，完善企业工资集体协商制度。构建赋予农民更多财产权利的实现机制，逐步建立农村产权流转交易市场。推动承包土地的经营权向金融机构抵押融资，探索集体资产股权进入市场。

提高人民对教育的满意度。完善现代教育体系，实施学校管办评分离制度。实施学前教育惠民扩优工程，促进教育公平。推动义务教育均衡发展，扩大优质教育资源。改革高中学校发展机制，整合优化资源配置。深化职业教育改革，助推转型升级。深化招生考试制度改革，减轻学生课业负担。畅通社会办学体制机制，促进民办教育规范发展。扩大高等教育合作办学，推进与澳门科技大学合作办学，加快推动省市联合培养研究生工作。优化师资队伍，推动校长教师交流。建设平安校园，保障师生安全。

提高市民健康水平。加快区域医疗卫生服务信息平台建设，落实新一轮医改政策。促进社区卫生服务中心实体化，启动“医联体”试点，完善基层医疗卫生机构网络。推广平价医疗服务，开展“治未病”健康工程，推进健康城市建设。鼓励社会办医，加快民营医疗机构发展，稳妥推进公立医院改革。加强社区体育设施建设，创建全民健身示范城市。

繁荣文化事业。健全公共文化服务体系，加大文化惠民力度，建设文化强市。巩固全国文明城市创建成果，深入开展全民修身行动。完善市镇村三级文化设施网络，推进中山纪念图书馆、139文化街区建设。丰富历史文化名城内涵，弘扬孙中山精神。提高文化开放水平，鼓励社会力量参与文化建设。培育一批骨干文化企业，提升游戏游艺、灯饰、红木家具等特色产业文化价值。

发展各类社会事业。落实上级计生政策，促进人口长期均衡发展。推进妇女事业全面进步和儿童健康成长。完善退役士兵安置制度和优抚对象优待政策，开展社会化拥军。整合外事侨务资源，统筹推进对外合作交流。做好对口帮扶潮州和扶贫工作。做好民族宗教、统计、人防、地震、国防建设、档案、地方志等工作。

办好十件民生实事。经过广泛吸纳民意，今年继续集中力量办好十件民生实事。一是进一步改善空气质量。改造（含拆除）锅炉370台，淘汰“黄标车”7000辆。年内建成黄标车限行电子执法系统。施工场地安装监控视频16个，加强扬尘监管，严格落实施工现场围挡、砂土覆盖、路面硬化、拆除工程洒水、出车冲洗“五个100%”。建立空气质量监测预警系统。二是实施城市畅通工程。实施道路改造工程，勤学路、东华路完成主体改造工程；动工改造康华路、景观北路、城桂路（孙文公园—南外环段）和博爱路景观路下穿地道；整治北外环南侧8.6公里；岐江桥、光明桥恢复人车通行；完成105国道中山细滘大桥至沙朗段10座跨线桥工程和5个地下人行通道建设。实施交通管理畅通工程，加强道路施工管理，治理改善出行高峰拥堵点10处。实施智能交通三期工程，改造升级城区65个路口交通信号系统。规范城市道路停车秩序管理，增设路内停车泊位1100个。规范改造城区9条主干道路的指路标志，维护完善城区道路交通标线。三是市镇两级设食品快检机构。市食品药品检验检测机构投入使用，在每个镇区都建立食品综合快检机构和水产品快速检测点。在市镇两级农贸市场设立市民自行申请食品质量检测服务点72个，食品安全投诉点72个。四是绿化工程覆盖城乡。建成或改造城镇道路绿化100公里、岐江河绿化2公里，山地森林景观改造2000亩。创建森林家园5个，建成森林公园5个、湿地公园3个，完成翠亨新区起步区路网绿化工程。五是推进公交优先及绿色出行。首条快速公交示范线试运营。新建公交候车亭100个，新增新能源公交车180辆。为全市23万60岁以上新老中山人提供免费乘坐公交服务。实施“平安公交”工程，全市20个汽车客运站和17个公交枢纽站配备治安设施设备，严厉打击公交违法犯罪行为。新投放公共自行车6000辆，新建桩位3000个。开通公共自行车二维码手机查询系统。新增城市绿道50公里，中心城区绿道循环畅通。六是加大困难群体生活保障力度。全面实施困难群体重特大疾病医疗救助制度。为符合条件的I型糖尿病患者免费发放胰岛素。在两个镇区试点开展社区慢性病患者综合服务，为高血压、糖尿病等各种慢性病患者实施健康行为干预和跟踪随访。落实最低生活保障标准与价格上涨联动机制和自然增长机制，准时足额发放最低生活保障金。七是优化社区居家养老服务。新建社区居家养老服务中心（站）3个，优惠为800名老人提供上门家政服务。发展社工专业人才300人，设立社工站点达45个。八是创建场所“无三害”城市。创建全国首个文化娱乐服务场所“无三害”（毒、赌、黄）城市，文化娱乐服务场所内刑事治安案件和群众对“三害”举报案件数量大幅减少，基本消除“三害”。创建全国禁毒工作示范市，实现新滋生吸毒人数增长率、涉毒违法犯罪案件增长率低于全国和全省平均水平。禁毒宣传教育覆盖率、社区吸毒人员知情率、社区戒毒人员帮教率、本地吸毒人员管控率实现“4个100%”。九是建设社区体育公园。每个镇区整合建设2—3个社区体育公园示范点，建设全民健身服务站点24个。农民健身设施向自然村延伸，40个自然村建有全民健身场地。十是实现村（居）“一站式”服务全覆盖。新建77个村（居）社区综合服务大厅，为群众提供劳动社保、民政残联、人口计生等“一站式”服务。100台网上办事大厅自助终端进村（居），为市民提供网上审批、政务公开等便民服务。

各位代表！为政之要，贵在落实，重在行动。加强政府自身建设，是全面完成今年各项目标任务的重要保证。要深入开展群众路线教育实践活动，倡导实干的政风，以“逢山开路、遇水搭桥”的勇气，保持“踏石留印、抓铁有痕”的劲头，狠抓各项工作落实。一是建设法治政府。自觉接受人大法律监督、工作监督和政协民主监督，定期向市人大报告工作和向市政协通报情况，认真办理人大建议和政协提案，主动听取各民主党派、工商联、各人民团体和各界人士的意见建议。严格依照法定权限和法定程序行使职权，做好行政复议。二是建设阳光政府。推进政务公开和网络问政，制定政府部门行政处罚自由裁量权清单，依法规范权力运行流程，实现决策公开、服务公开、结果公开。三是建设高效政府。扩大政府绩效管理覆盖面，创新考核方式，落实首问责任制、限时办结制。狠刹文山会海，改进文风会风。加强调查研究，加大督查督办力度。四是建设电子政府。推动网上办事大厅向镇区延伸，建设企业法人网页和市民网页，增强个性化功能，行政审批事项网上办理率达40%，网上深化率达90%。五是建设清廉政府。强化行政监察和审计，加强对政府全口径预算审查监督。稳步推进领导干部经济责任审计，强化土地出让、工程招标、政府采购、产权交易、资金使用等领域的监管，保持惩治腐败高压态势。严格控制“三公”经费、楼堂馆所建设和人员编制增长。
